# Lithium protects dopaminergic cells from rotenone toxicity via autophagy enhancement

**DOI:** 10.1186/s12868-015-0222-y

**Published:** 2015-11-25

**Authors:** Lingling Hou, Nian Xiong, Ling Liu, Jinsha Huang, Chao Han, Guoxin Zhang, Jie Li, Xiaoyun Xu, Zhicheng Lin, Tao Wang

**Affiliations:** Department of Emergency, Central Hospital of Wuhan, Wuhan, Hubei China; Department of Neurology, Tongji Medical College, Union Hospital, Huazhong University of Science and Technology, Wuhan, Hubei China; Division of Alcohol and Drug Abuse, Department of Psychiatry and Harvard NeuroDiscovery Center, Harvard Medical School and Laboratory of Psychiatric Neurogenomics, McLean Hospital, Belmont, MA USA

**Keywords:** Lithium, Autophagy, SH-SY5Y Cells, Parkinson’s disease

## Abstract

**Background:**

Previous studies have indicated that enhancement of autophagy lysosome pathway may be beneficial for Parkinson’s disease (PD), in which aberrant accumulation of aggregated/misfolded proteins and mitochondrial dysfunction are considered as crucial pathogenesis. Recently, a number of studies have suggested the neuroprotective effects of lithium in models of several neurodegenerative diseases including PD. However, the exact mechanisms underlying this neuroprotection remain unclear. In our study, rotenone-exposed SH-SY5Y cells were used as an in vitro parkinsonian model to assess the autophagy-enhancing effect of lithium and the underlying mechanisms were further investigated.

**Results:**

Similar to the common used autophagy enhancer rapamycin (Rap, 0.2 μM), lithium (LiCl, 10 mM) significantly recovered the shrinkage of SH-SY5Y cells, and alleviated rotenone-induced cell apoptosis, mitochondrial membrane potential reduction and reactive oxygen species accumulation. Furthermore, the protective effects induced by LiCl were partially blocked by the co-treatment of autophagy inhibitors such as 3-methyladenine (3-MA, 10 mM) or chloroquine (CHL, 10 μM). Moreover, 3-MA or Chl suppressed LiCl-induced autophagy in the immunoblot assay. In addition, the co-localization of LC3 and mitochondria and the preservation of mitochondrial function within LiCl-treated cells were observed, confirming that the damaged mitochondria were cleared through autophagy (mitophagy).

**Conclusions:**

These findings suggested that lithium exerted neuroprotection against rotenone-induced injuries partially through the autophagy pathway. Pharmacologically induction of autophagy by lithium may represent a novel therapeutic strategy as a disease-modifier in PD.

## Background

Parkinson’s disease (PD), a chronic and progressive neurodegenerative disease, is manifested as motor symptoms such as tremor, bradykinesia, rigidity and postural instability, as well as non-motor symptoms such as depression, apathy, sleep disorders and erectile dysfunction [1]. The hallmark pathological signs of PD include the degeneration of dopaminergic (DA) neurons in the substantia nigra and the formation of α-synuclein+ protein aggregates which are called Lewy bodies or Lewy neurites in the remaining DA neurons. Although treatments that slow or halt the disease process are currently unavailable, manipulating protein aggregation seems to have great potential for disease-modification [1].

There are two important mechanisms for protein degradation in the mammal cells, detailed as the autophagy-lysosome pathway (ALP) and the ubiquitin-proteasome system (UPS). Previous studies have demonstrated that dysfunction of these two systems plays vital roles in the pathogenesis of PD [2–5]. The function of UPS is limited by the core volume, and upregulated proteasome activity only leads to non-selective degradation of key short-lived intracellular regulators, resulting in detrimental consequences, such as cancer in the case of tumor suppressor p53 [1]. In contrast, ALP degrades entire organelles such as mitochondria and large membrane proteins which fail to recycle by UPS [4, 6]. Thus, enhancement of autophagy might be a novel strategy for the treatment of protein accumulation-related diseases, such as PD.

The commonly used strategy for inducing autophagy is employing rapamycin, an inhibitor of the mammalian target of rapamycin (mTOR) [7, 8]. Previous studies have revealed that rapamycin exerts neuroprotective effects in several models of neurodegenerative diseases such as Alzheimer’s disease (AD) and PD through autophagy enhancement [9–11]. However, since mTOR plays an important role in regulating a variety of basic cellular processes, long-term use of rapamycin may cause some deleterious consequences, including cell over-growth and proliferation [12]. It is crucial to explore an autophagy enhancer which is safe, clinical-relevant and easy to administrate.

Unlike rapamycin, lithium (LiCl) induces autophagy via an mTOR-independent pathway. By inhibiting the inositol monophosphatase (IMPase), LiCl ultimately results in the reduction of free inositol cellular level [13–16]. A recent study has indicated that LiCl suppressed Aβ pathology by inhibiting the translation of protein in a Drosophila model of AD [17]. Furthermore, LiCl protected against paraquat-induced neurotoxicity by NRF2 activation and miR-34a inhibition in SH-SY5Y cells [18]. Moreover, our previous studies have suggested that LiCl might be neuroprotective in rotenone-induced SH-SY5Y cells and MPTP-lesioned mice though autophagy enhancement [19, 20]. However, another study have indicated that LiCl was unable to alleviate the degeneration of DA neurons induced by 6-OHDA, suggesting that GSK3 was minimally involved in the neurodegeneration [21]. Thus, it remains unclear whether or not LiCl exerts neuroprotective effects and by which pathway LiCl is involved in the neuroprotection.

In this study, rotenone-exposed SH-SY5Y cells were used as an in vitro model of PD, and cell viability, activation of apoptosis, oxidative stress and mitochondrial dysfunction were assessed respectively [3, 22, 23, 24]. Rapamycin was used as a positive control for induction of autophagy. The neuroprotective effects of LiCl on the rotenone-induced cellular model as well as the underlying mechanisms were further explored.

## Results

### LiCl increased cell viability upon rotenone exposure in SH-SY5Y cells

**Fig. 1:**
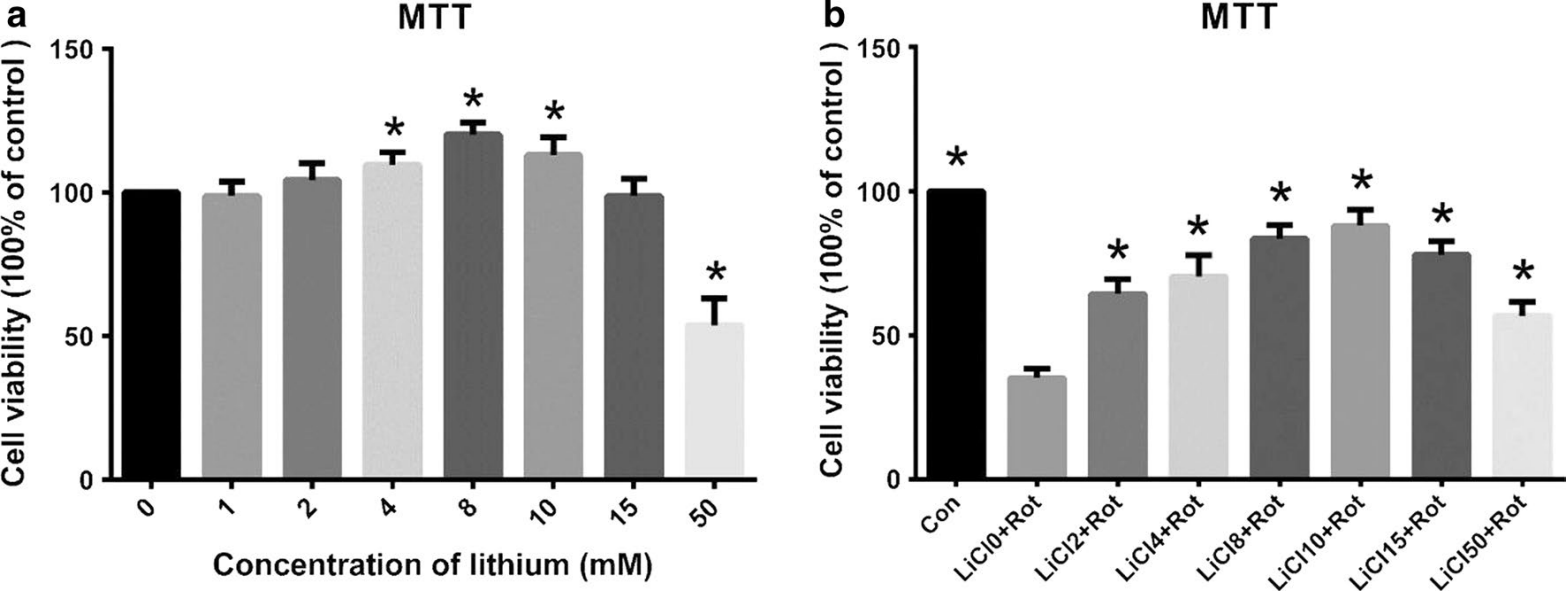
Lithium protected against rotenone-induced cytotoxicity in SH-SY5Y cells. Cell viability was measured by MTT assay after treatment of SH-SY5Y cells with lithium for 24 h at indicated concentrations with or without rotenone exposure. **a** Lithium exposure (0–15 mM) had no negative influence on the cell survival of SH-SY5Y cells under normal culture condition. **b** Lithium dose-dependently protected SH-SY5Y cells from rotenone-induced cytotoxicity. *P < 0.05 as compared with control (**a**) or LiCl 0 + Rot-treated cells (**b**) (n = 6 in each group)

We first examined whether or not LiCl affected cell survival under normal culture condition. MTT analysis indicated that in the range of 1 mM to 15 mM, LiCl did not significantly affect the viability of SH-SY5Y cells, while the concentration of 50 mM LiCl directly caused 48.3 ± 5.37 % reduction of MTT absorbance. When treated with rotenone for 24 h, rotenone decreased cell viability by 67.56 % compared with control group, and by 37.46, 32.61, 18.32, 13.68, 23.94 and 41.21 % in LiCl2 + Rot, LiCl4 + Rot, LiCl8 + Rot, LiCl10 + Rot, LiCl15 + Rot and LiCl50 + Rot groups, respectively (Fig. 1b). LiCl with a dose range from 2 mM to 50 mM could alleviate rotenone-induced cell death. Moreover, the most significant cytoprotection of LiCl was observed at the concentration of 10 mM (Fig. 1b). Therefore, we chose the concentration of 10 mM for the subsequent tests.

### LiCl prevented from rotenone-induced cell injury

**Fig. 2:**
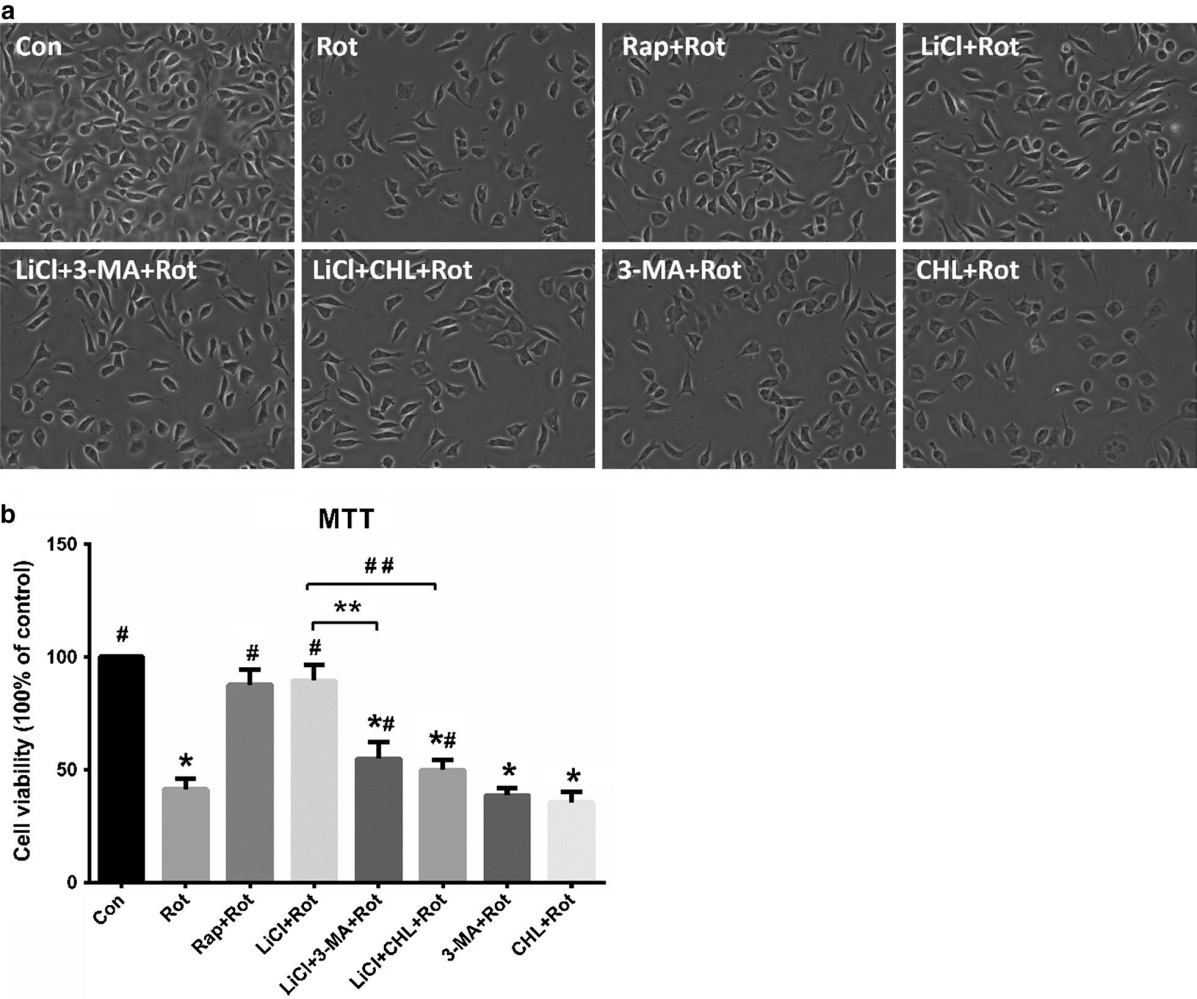
Co-treatment with autophagy inhibitors attenuated neuroprotection of lithium and increased vulnerability of SH-SY5Y to rotenone. Cells were pretreated with autophagy-related drugs for 24 h followed by rotenone exposure for another 24 h. The morphology changes of cells were shown in (**a**).The cell viability was measured by MTT assay (**b**) or Annexin-V/PI staining by flow cytometry (Fig. 3a). *P < 0.05 versus control; ^#^ *P* < 0.05 versus rot alone; **P < 0.05 as compared with LiCl + Rot group; ^##^ *P* < 0.05 as compared with LiCl + Rot group (n = 6 in each group)

**Fig. 3:**
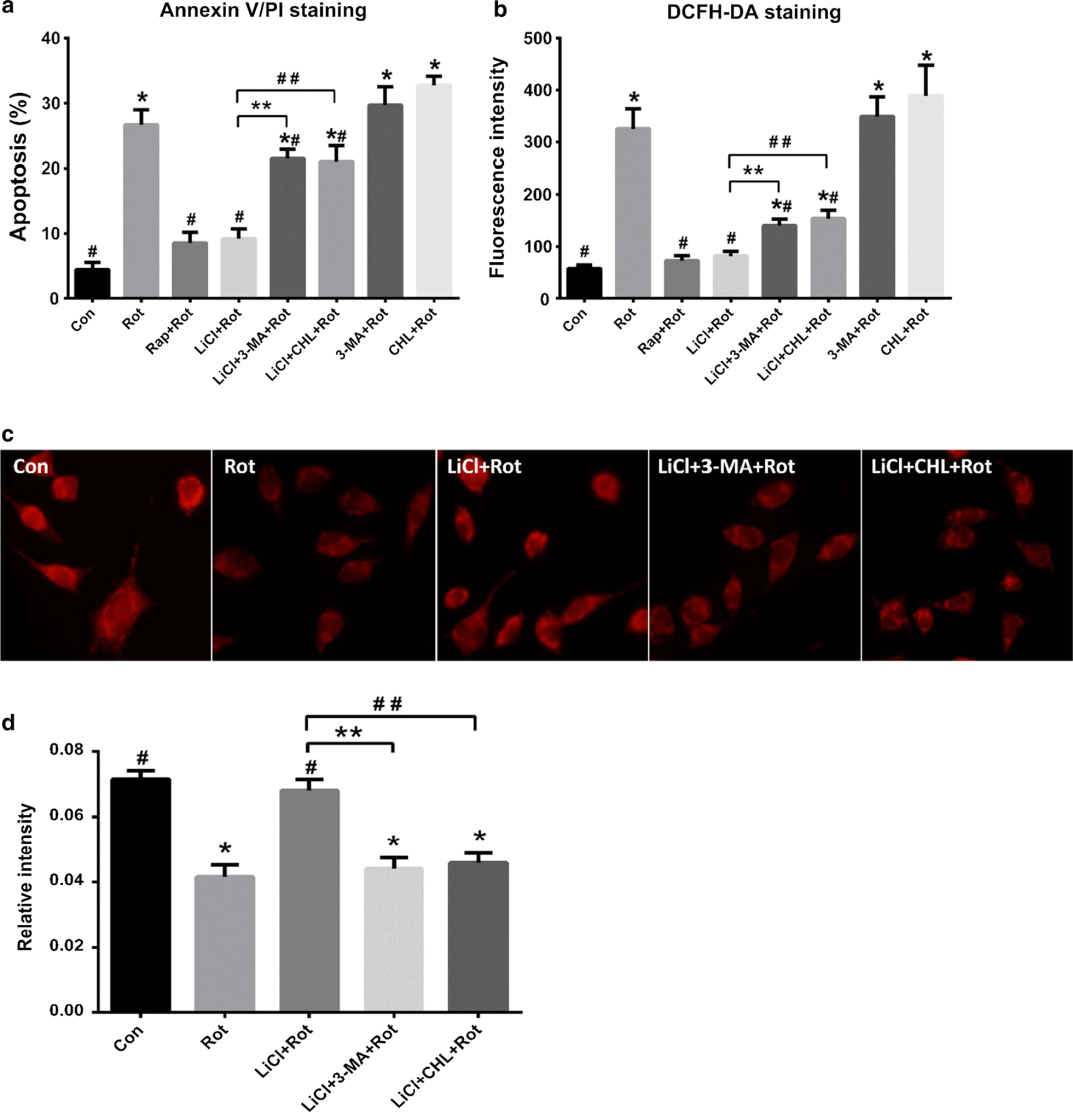
Lithium attenuated rotenone-induced dysfunction of mitochondria. Cells were pretreated with autophagy-related drugs for 24 h followed by rotenone exposure for another 24 h. The rate of cell apoptosis was measured by Annexin-V/PI staining (**a**) and the fluorescence intensity of DCFH was measured by flow cytometry in all groups (**b**). MitoTracker red CMXRos at a final concentration of 500 nM was used to visualize the mitochondrial transmembrane potential (**c**) and the relative fluorescence intensity was expressed (**d**). *P < 0.05 as compared with Con group; ^#^ *P* < 0.05 versus Rot alone, **P < 0.05 as compared with LiCl + Rot group; ^##^ *P* < 0.05 as compared with LiCl + Rot group (n = 6 in each group)

First, LiCl-induced neuroprotection was revealed by cell morphology. SH-SY5Y cells under normal culture condition grew into a neuron-like phenotype with extended neuritis. Exposure to rotenone (200 nM) for 24 h resulted in shortening of neurites and shrinking of the cell bodies, which was inhibited by rapamycin or LiCl pretreatment (Fig. 2a). However, the rehabilitative effect of LiCl was partially blocked when cells were co-treatment with the autophagy inhibitors 3-MA or CHL. The MTT assay indicated that rotenone significantly decreased cell viability by ~60 %, while pretreatment with rapamycin or LiCl significantly attenuated the toxic effects induced by rotenone. (Figure 2b). Such rescue of cell viability was partially attenuated by simultaneously pretreatment of 3-MA or CHL. Similar results were observed from cell apoptosis analysis (Fig. 3a). Rotenone exposure led to an increase of cell apoptosis, which was discounted by LiCl or rapamycin pretreatment, while the protective effect of LiCl was partially weakened by 3-MA or CHL, suggesting that the neuroprotective effect of LiCl may be related to autophagy induction (Fig. 3a).

### LiCl protected against rotenone-induced mitochondrial dysfunction

The SH-SY5Y cells in negative control showed accumulation of red fluorescence and extended neurites, while rotenone exposure led to reduced red fluorescence with condensed nuclei and cell shrinkage, indicating a collapse of MMP with exposure to rotenone (Fig. 3c). The reduction in MMP meant a lower capacity to produce ATP. Then the cells pretreated with LiCl significantly inhibited the decrease of fluorescence intensity, indicating that LiCl prevented rotenone-induced mitochondrial injury. In one hand, mitochondrial dysfunction triggered ROS formation, on the other hand, accumulated ROS could in turn cause oxidative damage of mitochondria. To confirm the neuroprotective effect of LiCl on mitochondria, we measured the levels of ROS in different groups. Rotenone evoked obvious production of ROS, and LiCl indeed alleviated the overgeneration of ROS. However, this protective effect of LiCl was partially attenuated when cells were simultaneously pretreated with 3-MA or CHL (Fig. 3b). In addition, pretreatment of cells with CHL resulted in a higher level of ROS release than the rotenone group, indicating that CHL increased cellular vulnerability to rotenone toxicity and mitochondrial dysfunction.

### LiCl enhanced autophagy in SH-SY5Y cells

**Fig. 4:**
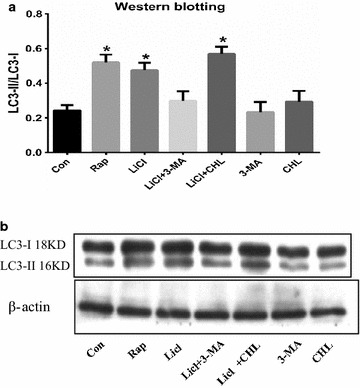
Co-treatment with autophagy inhibitors depressed lithium induced autophagy in SH-SY5Y cells. The cells were treated with autophagy-related drugs at various concentrations for 24 h. The induction of autophagy was determined by measuring the LC3 protein levels using immunoblotting assay with antibody against LC3 (**b**). β-actin was used as an equal loading of proteins. The ratio of LC3B-II/I was evaluated by densitometric analysis and data were expressed (**a**). *P < 0.05 as compared with Con group (n = 6 in each group)

In immunoblot assay, the ratio of LC3-II/I with LiCl pretreatment was increased by ~84 % when compared to the vehicle control, similar tendency to that of the rapamycin, while the ratio was slightly decreased with co-treatment of LiCl and 3-MA. LC3 expression was highest in the group with co-treatment of LiCl and CHL. By disrupting the pH of acidic vesicles, CHL prevented the fusion process of lysosomes and autophagosomes, and ultimately elevated the cellular burden of autophagosome accumulation [25]. Thus, the accumulation of LC3-II, a classic marker of autophagosome, suggested either that autophagy has been stimulated and/or that the maturation of autophagosomes has been blocked (in the case of the addition of CHL) [26]. Our immunoblot assay indicated that autophagy was up-regulated during LiCl or rapamycin exposure, and such induction of autophagy was attenuated when cells were treated with 3-MA or CHL simultaneously (Fig. 4), suggesting that the protective effect of LiCl was related to the induction of autophagy.

### Damaged mitochondria were eliminated via autophagy

**Fig. 5:**
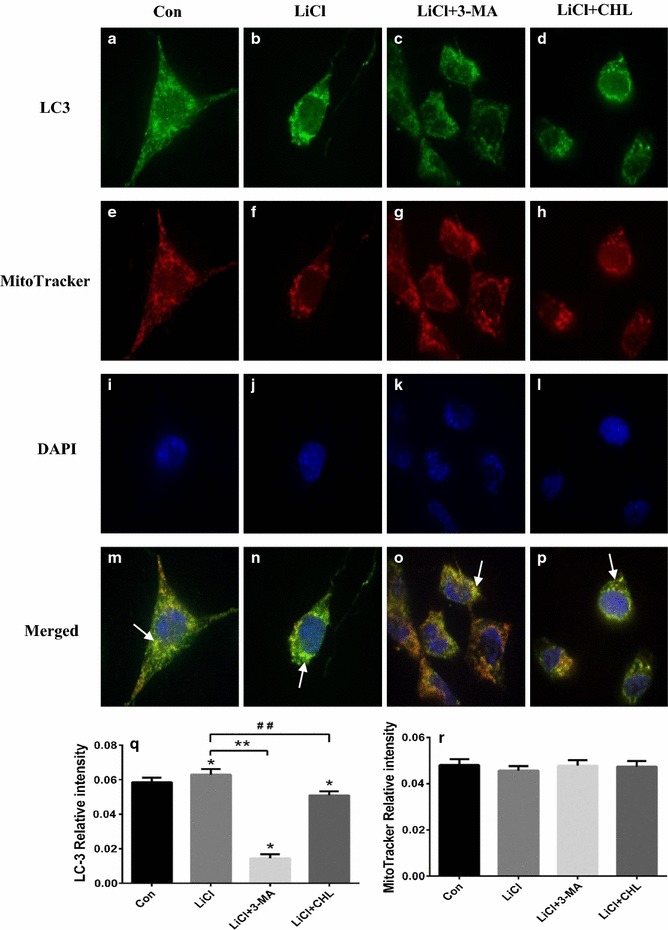
Lithium promoted degradation of mitochondria via autophagy. Cells exposed to lithium with or without autophagy inhibitors were incubated with anti-LC3 antibody (1:100) and MitoTracker Red to visualize the co-localization (*yellow immunofluorescence*) of LC3 (**a**–**d**) and mitochondria (**e**–**h**) in SH-SY5Y cells at ×400 magnifications under a confocal fluorescence microscope, and the nuclei were stained by DAPI (**i**–**l**). The co-localization of autophagosomes and mitochondria was marked using *white arrows* (**m**–**p**). The relative intensity of LC3 (**q**) and mitochondria were expressed (**r**). *P < 0.05 as compared with Con group; **P < 0.05 as compared with LiCl + Rot group; ^##^ *P* < 0.05 as compared with LiCl + Rot group (n = 6 in each group)

To detect the association between autophagy induction and the restored mitochondrial function, further immunostaining analysis without rotenone exposure was performed. The results showed that LiCl treatment contributed to a punctuate pattern of LC3 fluorescence in the cytoplasm of SH-SY5Y cells, while it led to a diffuse distribution of LC3 in the absence of LiCl. Additionally, this effect could be partially attenuated by 3-MA or CHL co-treatment (Fig. 5). As shown in the Fig. 5r, there is no statistical difference between these groups regarding the normalized MitoTracker Red fluorescence in the cytoplasm. However, 3-MA or CHL co-treatment significantly reduced the LC3 aggregation induced by LiCl (Fig. 5q). Co-localization of MitoTracker Red and LC3 indicated that the turnover of mitochondria might be within autophagolysosomes.

## Discussion

As a mood stabilizer, LiCl is traditionally used to treat bipolar disorder [27]. Recent research has focused on its neuroprotective potential and regarded it as a candidate agent for disease-modifying therapy in several neurodegenerative diseases [28–30]. The neuroprotective effect of LiCl may rely on autophagy induction. In our study, human SH-SY5Y cells exposed to rotenone were used as an in vitro model of PD to explore the effects of LiCl on rotenone neurotoxicity and its underlying mechanisms. These findings indicated that LiCl could attenuate cell apoptosis, inhibit ROS production and MMP decrease induced by rotenone. Moreover, the protective effects were partially blocked upon pretreatment with autophagy inhibitors such as 3-MA or CHL.

Compared with control group, the ratio of LC3-II/I was elevated by LiCl treatment, showing that LiCl induced autophagy in SH-SY5Y cells. However, this autophagy-enhancing effect was partially blocked by 3-MA, a widely used inhibitor of autophagy. As CHL could enhance the cellular burden of autophagosomes which were manifested as the accumulation of LC3-II protein, the increased ratio of LC3-II/I by chloroquine treatment indicated that the maturation of autophagosomes has been blocked, representing an inhibition of the autophagy flux [25].

As a mitochondrial complex I inhibitor, rotenone is extensively used for modelling PD in recent years. In this study, rotenone induced mitochondrial dysfunction as evidenced by the increased production of ROS and the decreased MMP, which may lead to the activation of the mitochondrial apoptotic pathway [31–33]. Mitochondria have been proposed as an important link among risk factors, and eliminating injured mitochondria would be critical to prevent cells from a series of pro-apoptotic cellular responses. Autophagy is the only process by which organelles such as mitochondria can be degraded. Previous studies indicated that inhibited autophagy was accompanied by accumulated mitochondrial mass [34–36]. Thus, we hypothesized that pretreatment with autophagy-enhancing agents like LiCl might be able to reduce mitochondrial load. As expected, LiCl reduced rotenone-induced apoptosis, ROS production and MMP reduction, exerting similar neuroprotective effects to another autophagy enhancer rapamycin. To further investigate whether the neuroprotection of LiCl was autophagy-dependent, two chemical suppressors of autophagy 3-MA and CHL were used. The converse results were observed in cells treated simultaneously with 3-MA or CHL, corresponding to the reduced ratio of LC3II/I which suggested that the protective effect of LiCl could be partially blocked as autophagy was inhibited. Then we observed that the mitochondria fluorescence was well merged with the LC3 fluorescence in the cytoplasm of LiCl-treated cells when compared to those treated with autophagy inhibitors or vehicle control. This further supported an elimination of mitochondria via autophagy. As the sole known mechanism for mitochondrial turnover, autophagy serves to preserve the balance between organelle biogenesis and their clearance, and engages in cross-talk with mitochondria and oxidative stress [37].

Our study have indicated that LiCl alleviated rotenone-induced cell apoptosis and ROS production through autophagy induction, which was consistent with previous findings that induction of autophagy could protect against pro-apoptotic insults by reducing mitochondrial load [16, 38, 39]. Scola et al. also found that lithium prevented rotenone-induced changes in mitochondrial complex I function, cell death and changes to DNA methylation and hydroxymethylation [39]. These findings let us have a more balanced view on the effects of lithium. Since lithium is known to pass the blood–brain barrier and has already been approved by FDA for decades for the treatment of bipolar disorder [40], we could reasonably speculate that by inducing autophagy, lithium may have great potential as a novel therapy for PD, although the possibility requires further investigation.

## Conclusions

Taken together, these findings indicated that lithium was able to attenuate cell apoptosis, inhibit ROS production and MMP decrease induced by rotenone in SH-SY5Y. Moreover, autophagy enhancement was involved in this neuroprotection as the effects of lithium were partially blocked upon pretreatment with autophagy inhibitors. Pharmacologically induction of autophagy by lithium may raise a new hope for novel disease-modifying strategies in PD.

## Methods

### Cell culture and treatment

Human neuroblastoma SH-SY5Y cells (ATCC, Manassas, VA, USA) were cultured in DMEM/F12 medium (Invitrogen, Carlsbad, CA, USA) at 37 °C with 5 % CO_2_ and 95 % air (v/v). The growth medium was supplemented with 10 % fetal bovine serum (Invitrogen). Rotenone (Sigma-Aldrich, St. Louis, MO, USA) and rapamycin (Sigma-Aldrich) were dissolved in dimethyl sulfoxide (DMSO) before diluted with the culture medium, and the final concentration of DMSO per well was less than 0.2 %. 3-methyladenine (3-MA) (Sigma-Aldrich) was dissolved by heating in ddH_2_O, while LiCl and chloroquine (CHL) were dissolved in phosphate buffered solution (PBS) before dilution with the culture medium. PBS and DMSO were added to the culture medium in negative control group (PBS for 24 h following DMSO for another 24 h, “Con-group”). The final concentrations of Rap [20, 41], LiCl [20, 42], 3-MA and Chl [20, 43] were 0.2 μM, 1–50 mM, 10 mM and 10 μM, respectively. Rotenone (200 nM) was given for 24 h to induce cell damage. For MTT assay, apoptosis analysis, mitochondrial membrane potential (MMP) and reactive oxygen species (ROS) detection, SH-SY5Y cells were pretreated with vehicle (PBS), Rap, LiCl, LiCl + 3-MA, LiCl + CHL, 3-MA, CHL, respectively, for 24 h, and then rotenone for another 24 h. For assessing microtubule associated protein 1 light chain 3 (LC3) expression in immunostaining and immunoblotting, the cells were only treated with vehicle, Rap, LiCl, LiCl + 3-MA, LiCl + CHL, 3-MA, CHL, respectively, for 24 h.

### Measurement of cell viability

Cell viability was detected in MTT assay by a microplate reader (Bio-Rad, Hercules, CA, USA) [22, 44, 45]. Briefly, SH-SY5Y cells were seeded at a density of 1 × 10^4^ cells per well in 96-well plates. When exposed to different treatments for the indicated times duration, 20 µL of MTT solution (5 mg/mL, Sigma-Aldrich) were added to each well for 4 h to allow the formation of purple formazan crystal. Then, 100 μL of the solubilization reagent was added into each well and spectrophotometrically measured for absorption at λ 570 nm.

### Apoptosis analysis

The FITC-conjugated Annexin V (Annexin V, Sigma-Aldrich) and propidium iodide (PI, Sigma-Aldrich) double-staining followed by flow cytometry (BD, Franklin Lakes, NJ, USA) assessment was employed to analyze the rates of cell apoptosis [22, 44, 45]. Cultivated termination, 2 × 10^6^ cells were harvested with 0.25 % trypsin and washed with PBS, and then incubated with Annexin V-FITC (apoptosis detection kit, Sigma-Aldrich) and 20 μg/mL PI (10 μL) for 10 min at 37 °C in the darkness. Then FITC and PI signals were measured with a BD-LSR flow cytometer using the CellQuest software. Data were analyzed by FSC express version 3.0 (De Novo Software, Los Angeles, CA, USA). The apoptosis rate = [Annexin V(+)PI(−) cells + Annexin V(+)PI(+) cells]/total cell × 100 %.

### Measurement of reactive oxygen species

Fluorescent probe 2,7-dichlorofluorescein diacetate (H_2_DCFDA) was used to measure the intracellular ROS [22, 44, 45]. The generation of dichlorofluorescein (DCF), an oxidized form of H_2_DCFDA, is proportional to intracellular ROS levels. At the termination of medication, cells were harvested, resuspended in PBS and immediately incubated with 10 μM H_2_DCFDA (Sigma-Aldrich) for 30 min at 37 °C in the darkness. After washed, the samples were detected by a FACScan flow Cytometer (BD). Data were analyzed by FSC express version 3.0 (De Novo Software, Los Angeles, CA, USA).

### Detection of mitochondrial membrane potential

Since MitoTracker probe could pass through the plasma membrane and accumulate in mitochondria [22, 46], assessment of MMP was performed using MitoTracker Red CMXRos (Invitrogen). After harvested and resuspended with PBS, cells were cultured with MitoTracker Red CMXRos (500 nM) for 30 min at 37 °C in the darkness, washed with PBS twice and then the nuclei were stained with DAPI (Invitrogen). The MMP level was visualized quantitatively as red fluorescence, while the DNA could be simultaneously observed as blue fluorescence.

### Autophagy detection in immunoblotting analysis

Autophagy level was detected by a specific marker protein, the autophagosomal membrane form of microtubule-associated protein 1 light chain 3 (LC3) [5], which has two forms, LC3-I (18 KD, a cytoplasmic form) and LC3-II (16 KD, a cleavage form). The conversion of LC3-I into LC3-II (LC3-II level compared to LC3-I level) was indicative of autophagic activity. Higher ratio of LC3-II/LC3-I means higher autophagic activity. After harvested and washed by cold PBS, cells were directly lysed in 100 mL of 2× SDS sample buffer containing protease inhibitors and boiled for 5 min [22, 44, 45]. Then total protein concentration was determined by a BCA kit (Pierce, Rockford, IL, USA). Equal amounts of proteins (40 mg) were separated by SDS–polyacrylamide gel electrophoresis and transferred to polyvinylidene difluoride membrane (Pierce). The membranes were then washed three times with PBS, blocked with 5 % dry milk in Tris-buffered saline for 45 min and incubated with primary antibodies directed against LC3 (1: 1000) and β-actin (Santa Cruz, Santa Cruz, CA, USA) overnight at 4 °C. Following three 10-min washes in tris-buffered saline tween-20, the membranes were incubated with the corresponding secondary antibody conjugated with HRP at room temperature for 1 h. Antibody binding was visualized using enhanced chemiluminescence ECL-Plus (Amersham Pharmacia, Piscataway, NJ, USA). Blots were quantified using analysis system (Quantity One, Hercules, CA, USA).

### Visualization of the autophagic degradation of mitochondria (mitophagy)

When harvested, SH-SY5Y cells were fixed with 4 % paraformaldehyde (Amresco llc, Solon, OH, USA) at 4 °C for 10 min, washed with PBS and permeabilized with 0.5 % Triton X-100 for 2 min, then incubated with primary antibody, the monoclonal anti-LC3 antibody (1:100) overnight at 4 °C followed by the secondary antibody, a fluorescein isothiocyanate conjugated goat anti-rabbit antibody (1:60, Sigma-Aldrich) for 2 h at room temperature. Cells were observed by using a confocal microscope (Olympus, Tokyo, Japan) and then images were analyzed by Image-Pro Plus 6.0 software package (Bethesda, MD, USA) [20].

### Statistical analysis

Statistical analyses were carried out using SPSS version 20.0 for Windows (IBM Corporation, New York, USA). All values were expressed as mean ± SD (n = 6 in each group). The statistical significance of differences between groups was evaluated using a one-way ANOVA followed by Fisher’s least significant difference t test, as a normal distribution in all groups was given. *P* value of <0.05 was considered as statistically significant.

## Availability of supporting data

The data set supporting the results of this article is included within the article.

## Abbreviations

ALP: autophagy lysosome pathway
ROS: reactive oxygen species
CHL: chloroquine
3-MA: methyladenine
DA: dopaminergic
UPS: ubiquitin–proteasome system
mTOR: mammalian target of rapamycin
IMPase: inositol monophosphatase
Rot: rotenone
MMP: mitochondrial membrane potential
DMSO: dimethyl sulfoxide
PBS: phosphate buffered solution
H_2_DCFDA: 2,7-dichlorofluorescein diacetate
DCF: dichlorofluorescein
LC3: microtubule associated protein 1 light chain 3
